# A normalized template matching method for improving spike detection in extracellular voltage recordings

**DOI:** 10.1038/s41598-019-48456-y

**Published:** 2019-08-19

**Authors:** Keven J. Laboy-Juárez, Seoiyoung Ahn, Daniel E. Feldman

**Affiliations:** 1 Department of Molecular and Cell Biology and Helen Wills Neuroscience Institute, University of California Berkeley, Berkeley, CA 94720 USA; 2 Present Address: Department of Organismic and Evolutionary Biology and Center for Brain Science, Harvard University, Cambridge, Massachusetts USA

**Keywords:** Sensory processing, Neurophysiology

## Abstract

Spike sorting is the process of detecting and clustering action potential waveforms of putative single neurons from extracellular voltage recordings. Typically, spike detection uses a fixed voltage threshold and shadow period, but this approach often misses spikes during high firing rate epochs or noisy conditions. We developed a simple, data-driven spike detection method using a scaled form of template matching, based on the sliding cosine similarity between the extracellular voltage signal and mean spike waveforms of candidate single units. Performance was tested in whisker somatosensory cortex (S1) of anesthetized mice *in vivo*. The method consistently detected whisker-evoked spikes that were missed by the standard fixed threshold. Detection was improved most for spikes evoked by strong stimuli (40–70% increase), improved less for weaker stimuli, and unchanged for spontaneous spiking. This represents improved detection during spatiotemporally dense spiking, and yielded sharper sensory tuning estimates. We also benchmarked performance using computationally generated voltage data. Template matching detected ~85–90% of spikes compared to ~70% for the standard fixed threshold method, and was more tolerant to high firing rates and simulated recording noise. Thus, a simple template matching approach substantially improves detection of single-unit spiking for cortical physiology.

## Introduction

Extracellular single unit recording is widely used to monitor activity of neuronal populations, determine sensory tuning and evaluate stability and plasticity of neural coding over time^1,2^. Because raw extracellular voltage signals incorporate spiking of many nearby neurons over background noise, spike sorting is used to isolate action potentials and assign them to putative single neurons via waveform clustering^3–5^. Thus, high quality in both spike detection and clustering algorithms are critical to accurately measure single unit activity.

Recent advances have significantly improved spike clustering procedures, greatly reducing the need of manual intervention and allowing recording of single unit activity in large neuronal populations over long periods of time^6–8^. Other work has improved spike detection relative to background electrical noise through the use of template-based filtering^9–12^, wavelet transforms^13–15^ and energy operators^16,17^. For review of spike-detection methods see refs^4,5^. Despite these advances however, spike detection is still mostly done with a fixed voltage amplitude threshold. Here we show that this method fails to detect spikes during spatiotemporally dense population neural activity in cerebral cortex. In primary whisker somatosensory cortex (S1), we show that this results in systematic spike detection errors that distort measurements of neural activity and sensory tuning. We developed a simple template matching and data-driven optimization algorithm that improves many of the spike detection errors common to the fixed voltage threshold approach. This underscores the importance of implementing model-based methods for separating spikes from background noise.

Standard spike detection consists of (i) setting a fixed voltage threshold, (ii) classifying any voltage fluctuation that crosses this threshold as a putative spike, and (iii) storing a brief segment of recorded voltage (termed a voltage clip) around each threshold crossing for subsequent spike clustering. To prevent a single spike from being detected multiple times as separate events, a brief shadow period is enforced in which spike detection is disabled for ~0.6 ms after the initial threshold crossing. Thus, the ability to detect spikes is directly determined by the voltage threshold and the shadow period. If the threshold is too stringent, low amplitude (yet potentially sortable) spikes will go undetected, which can cause entire single units to be missed, or cause the loss of a fraction of spikes from well-isolated single units whose mean spike amplitude is relatively near detection threshold. Conversely, if detection threshold is too low, more noise and non-sortable spikes will cross threshold, increasing the fraction of time spent in shadow period, and thus reducing detection of larger, sortable spikes^18^. Despite the strong dependence of overall spike sorting on the voltage threshold, the value of the voltage threshold is often chosen arbitrarily, e.g. as a fixed multiple of the standard deviation of the voltage signal on a given recording channel.

The number of legitimate spikes that occur during shadow periods — and therefore are missed in spike detection — depends on the temporal dynamics of local neuronal activity. The number of shadowed spikes will increase with both average firing rate and with temporally correlated firing of nearby neurons. Such spatiotemporally dense firing often occurs in topographically organized brain regions, such as primary sensory cortex, when an optimal sensory stimulus for the local population is presented^19–21^ and is important for coding sensory features^22–24^. As a result, more spikes are likely to be missed in response to locally preferred stimuli, which will distort measurements of neural tuning. To maximize spike detection under these conditions, an improved method of spike detection needs to be implemented. We developed a normalized template matching method (NTM spike detection) that uses a different type of threshold – waveform similarity to a candidate single-unit spike waveform, rather than spike amplitude – and applies a data-driven approach to optimize detection thresholds for each individual single units. We show that this approach reduces shadowed spikes and selectively increases detection of candidate spike waveforms relative to non-sortable spikes, yielding improved estimates of neural responsiveness and tuning in sensory cortex.

## Results

### The normalized-template-matching (NTM) method

**Figure 1:**
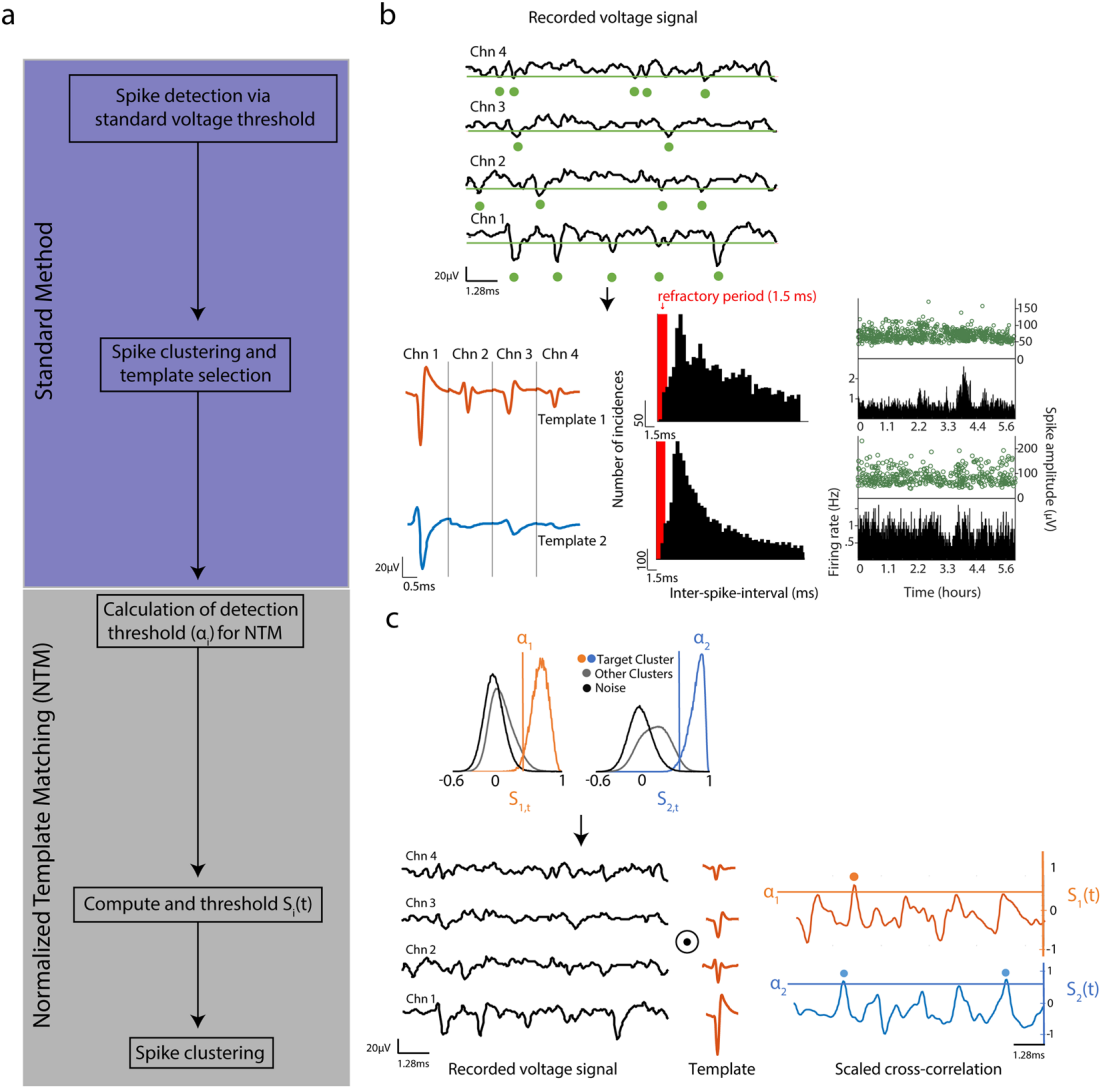
Normalized template matching algorithm for spike detection **(a)** Step-by-step description of normalized-template-matching (NTM). The standard method is an initial round of spike sorting with the usual fixed voltage threshold detection method. NTM is the second round of spike sorting where NTM is used for spike detection. **(b)** Graphical schematic of the standard method. Top, segment of extracellular voltage signal. Green line is the fixed threshold (usually 3 standard deviations below the mean) and dots denote threshold crossing events. Bottom, mean spike waveform of 2 example isolatable single-units. ISI distributions (in 0.5 ms bins) and spike amplitude and firing rate as a function of time are also shown. **(c)** Graphical schematic of NTM. Top, calculation of detection threshold for each template based on the spikes detected in the standard method. S_i,t_ is the cosine similarity between the template and a spike. Target cluster are spikes that were assigned to the single-unit of interest. Bottom shows the calculation of the scaled cross-correlation S_i_(t) for each template. Dots denote events that were classified as spikes.

Spike-sorting with the NTM method is a two-part process (Fig. 1a). First, an initial round of spike detection is performed using a standard fixed voltage threshold, and spike sorting is performed to cluster spike waveforms by their shape. The goal of this initial round is to identify mean spike waveforms of candidate, isolatable single-units. We term this initial round the ‘standard method’. This is followed by a second round where spike detection is performed via the NTM template matching method, and the goal is to maximize spike detection for these specific candidate single units. Detected spikes are then clustered by spike sorting, as in the standard method. We use one particular spike clustering method here, but NTM can be coupled with any clustering method.

### First step: the standard fixed-threshold method

In the initial spike detection procedure, we identified spikes by applying a fixed threshold to band-pass filtered voltage traces that were referenced to a common average across recording channels^25^ (see Methods). Any negative-going voltage transient that crossed the chosen threshold (usually 3 standard deviations below the mean) and did not fall in a shadow period was classified as a spike (Fig. 1b). A shadow period of 0.66 ms was enforced after each threshold crossing. This shadow period was chosen empirically, as the smallest period that prevented double-counting of individual spikes. This spike detection method is only sensitive to voltage amplitude not shape; background recording noise and spikes from non-isolatable units (multi-unit activity) can also pass threshold, be detected, and trigger a subsequent shadow period. Because a shadow period was used, both noise and multi-unit (non-sortable) activity can suppress detection of single-unit spikes.

After fixed-threshold spike detection, a semi-automated clustering algorithm was used to sort detected spike waveforms into clusters (putative single-units). We used an open source toolkit that performed the threshold-based spike detection, clipped 1.5-ms voltage segments around each non-shadowed threshold crossing, and then clustered clipped waveforms with a hierarchical clustering algorithm^26,27^. Briefly, clipped spikes were aligned with respect to their negative-going peak and over-clustered through recursive bisection. These clusters were then aggregated based on spike waveform similarity and inter-spike-intervals (ISI) while minimizing refractory period violations that are commonly assumed for single units. Final clusters were classified as isolatable single units by manual inspection using commonly used quality metrics^18^. These metrics were refractory period violations <0.5% of the total number of waveforms in the cluster, and <30% of spikes missing because their amplitude did not exceed the user-defined voltage threshold. The fraction of missing spikes was estimated from a Gaussian fit of the spike amplitude distribution; see Methods. These clusters were then used as candidate isolatable single units in the second step. Clusters that did not meet these single-unit quality metrics were considered ‘multi-unit clusters’, and were not further analyzed.

### Second step: NTM spike detection and re-clustering

We performed a second round of spike detection and sorting in which NTM was used to maximize spike detection for the candidate units identified in the first step. NTM detects spikes based on spike waveform similarity, not amplitude or absolute voltage threshold crossing. Waveform similarity is determined by calculating the cross-correlation between the extracellular voltage signal and the mean spike waveform (template) of each candidate single unit (Fig. 1c). We define the template for single unit *i* as μ_i_ = [μ_i,1_, μ_i,2_, …, μ_i,N_] where μ_i,c_ is the mean spike waveform of single unit *i* in electrode (or channel) *c*. In the data below, tetrode recordings were used, so N = 4. The waveform μ_i,c_ is a vector with *L* samples with *L* = f_s_k, where f_s_ is the sampling frequency and k is the user-defined time window of voltage over which the spike waveform is defined (in this study k = 0.0015 s; from 0.5 ms before to 1 ms after threshold crossing). Thus, μ_i_ is a 1 × (L*N) row vector built by horizontally concatenating mean spike waveforms across electrodes. Similarly, we define the extracellular voltage signal as a time-dependent row vector V(t) = [**v(t)**_**1**_, **v(t)**_**2**_, …, **v(t)**_**N**_], where **v(t)**_**c**_ is a segment of voltage signal on electrode *c* from sample *t* to sample *t* + *L*; i.e. **v(t)**_**c**_ = [v(t)_c_, v(t + 1)_c_, …, v(t + L−1)_c_]. Hence V(t) has the same dimensions as μ_i_. The cross-correlation between the signal V(t) and template μ_i_ is given by the expression:

$${C}_{i}(t)=V(t)\cdot {\mu }_{i}^{T}$$

where (∙)^T^ is the vector transpose. Thus the cross-correlation as a function of time is simply a sliding dot product between the voltage signal and the template. (Note that because of how V(t) is constructed, this dot product ‘slides’ through time, not recording channel). The dot product of these two vectors can be rewritten as:

$$V(t)\cdot {\mu }_{i}^{T}=\Vert V(t)\Vert \Vert {\mu }_{i}^{T}\Vert \,\cos (\theta )$$

where ||∙|| is the vector magnitude and θ is the angle between V(t) and μ_i_. Since ||μ_i_|| is constant, changes in C_i_(t) will only be due to changes in cos(θ) and ||V(t)||. Cosine of θ is a measure of similarity between the extracellular voltage segment and the template; a value close to 1 means that the two signals have a strong correlation in time (i.e. similar shape) because θ ≈ 0. ||V(t)|| is directly related to the energy of the extracellular voltage segment and will increase during epochs of high-amplitude voltage fluctuations. These high-amplitude voltage events can be caused by spiking of nearby neurons or electrical noise. Since the events of interest consist only of those with a similar shape to μ_i_ we remove the influence of the signal energy by scaling the cross-correlation C_i_(t) to:

$${S}_{i}(t)=\frac{C(t)}{\Vert V(t)\Vert \Vert {\mu }_{i}^{T}\Vert }=\,\cos (\theta )$$

Thus, the scaled cross-correlation S_i_(t), which we refer to as normalized-template-matching (NTM), is equivalent to a sliding cosine similarity between V(t) and μ_i_ (Fig. 1c).

After computing S_i_(t) we calculate a threshold α_i_ such that any point in time where S_i_(t) ≥ α_i_ is detected as a spike and stored as a voltage clip for clustering. An important difference between NTM and the fixed voltage threshold method is that we compute α_i_ through a data-driven approach rather than arbitrarily choosing a threshold relative to the noise floor. In this approach, we calculate the value of α_i_ through a receiver operating characteristic (ROC) curve such that the true positive detection rate (TP) of spikes from candidate units and correct rejections (CR) for multiunit activity and noise are maximized. For every single-unit *i* we compute the cosine similarity between each voltage clip and its template μ_i_:

$${S}_{i,t}=\frac{{E}_{t}\cdot {\mu }_{i}}{\Vert {E}_{t}\Vert \Vert {\mu }_{i}\Vert }$$

where E_t_ is a voltage segment that exceeded the amplitude threshold at time *t* and S_i,t_ is the cosine similarity between E_t_ and μ_i_. After spike clustering, the distributions of S_i,t_ for putative spikes that were classified as belonging to single unit *i* can be compared to the remaining putative spikes (Fig. 1c). The threshold α_i_ is chosen as the value of S_i,t_ that maximizes the percent correct classification of putative spikes belonging to single unit *i*, i.e. maximizes (TP + CR)/2. Importantly, voltage segments that don’t exceed the fixed voltage amplitude threshold (noise events) have a mean cosine similarity close to 0 that is separable from spikes from single units (Fig. 1c). NTM thus represents a nonlinear filtering operation that is invariant to multiplicative scaling of the voltage signal and output is bounded to ±1. This framework is similar to previous studies using linear filtering^9,10,28^ with the difference that NTM does not require the calculation and inversion of the noise covariance matrix (used for whitening the voltage signal), and thus represents a computationally simpler approach. A similar normalization operation was used in ref.^12^.

### NTM detects more spikes than the standard fixed-threshold method

As previously mentioned, standard spike detection algorithms only use voltage amplitude to detect spikes and are thus agnostic to spike waveform shape. NTM restricts spike detection to spikes from candidate isolatable single-units (Fig. 1b,c). We tested whether this improves spike detection rates for single units in primary whisker somatosensory cortex (S1) of anesthetized mice. Whisker deflections were applied to 9 different whiskers via computer-controlled actuators, so that whisker-evoked and spontaneous spiking could be assessed. Recordings were made using 32-channel silicon probes from layer (L) 2/3 and L4. Spike detection and sorting were performed on groups of 4 nearby recording pads, with each 4-channel group treated as an independent tetrode. Recordings were common average referenced, filtered at 0.3–8 kHz and sampled at 31.25 kHz (see Methods).

**Figure 2:**
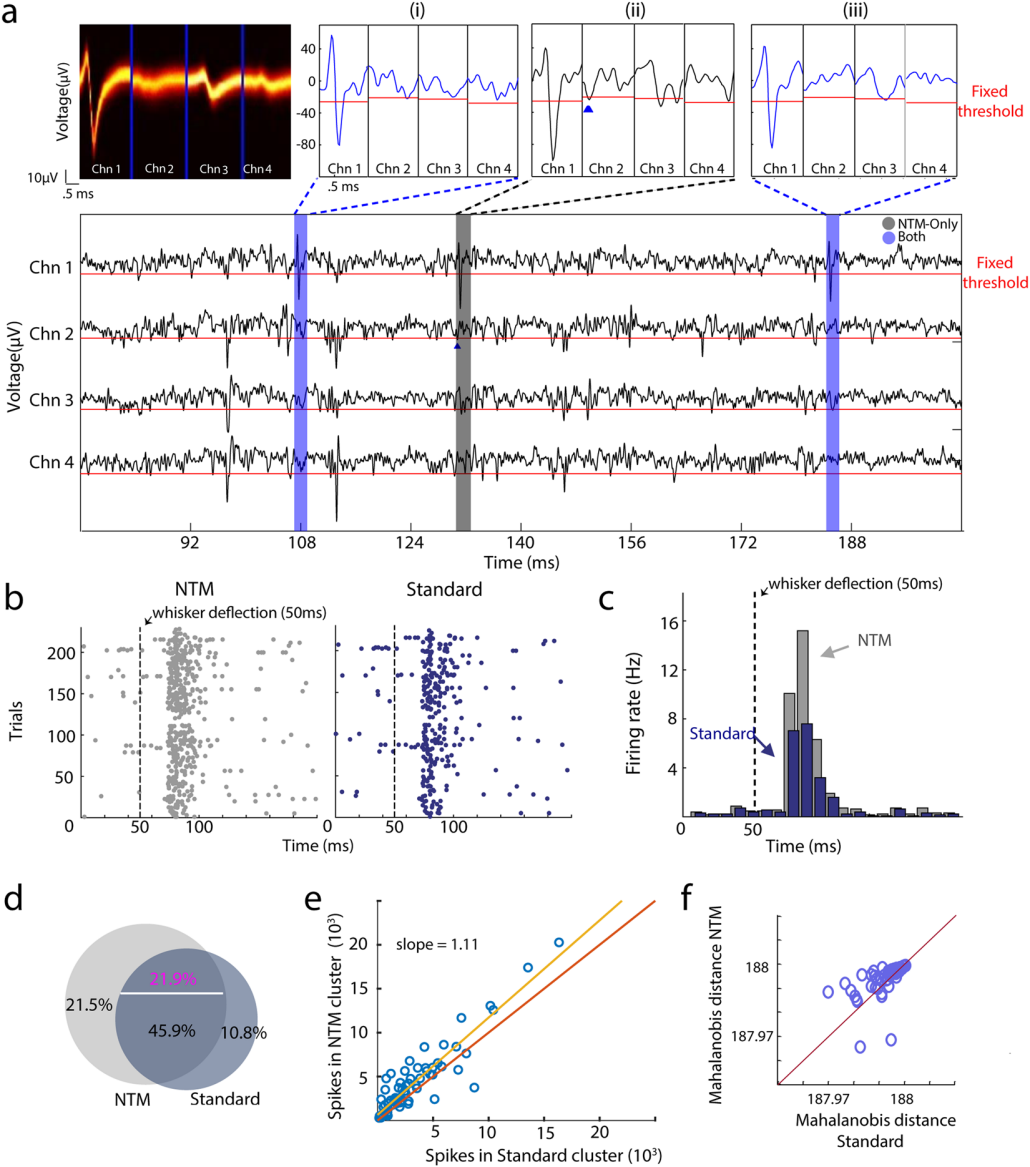
NTM detects more spikes than the standard fixed-voltage threshold method. **(a)** Example in which some spikes from a single unit were missed by the voltage threshold trigger but detected by NTM. Top left, distribution of spike waveforms belonging to the example single-unit. Bottom, segment of voltage signal from the 4 electrodes where the example single-unit was detected. Gray and blue regions indicate spike events from the example single unit that were detected only with NTM and with both spike detection methods respectively. Top right show individual spike waveforms. Triangle is a voltage threshold crossing event that suppressed spike detection. **(b)** Raster plot for an example single-unit using the standard and NTM spike detection methods. Black dash line is the onset of a whisker deflection. **(c)** Peri-stimulus-time-histograms (PSTHs) for the same example unit as (**b**) for NTM and standard spike detection. Bin size was 10 ms. The standard PSTH was shifted in time for clarity. **(d)** Venn diagram showing the mean percentage of spikes only detected with NTM, standard voltage threshold or both. Percentage of spikes that were detected by both methods but assigned to different units (misclassified) after clustering is shown in magenta. **(e)** Total number of spikes detected for each single unit with NTM vs. standard voltage threshold. Misclassified spikes were not included. **(f)** Mean Mahalonobis distance for each single-unit with NTM and the standard voltage threshold.

Figure 2a shows an example recording that illustrates how template matching can improve spike detection. First, standard spike detection was performed using a fixed voltage threshold, followed by the 0.66-ms shadow period, on each tetrode channel. Note that each channel has a different standard deviation and thus a different fixed threshold (red lines in Fig. 2a). Spikes (i) and (iii) were successfully detected and found to be part of an isolatable cluster, whose mean waveform across the 4 tetrode channels is shown as a spike density graph in the upper-left panel of Fig. 2a. But spike (ii) was censored by the shadow period of preceding voltage crossing events (triangle in inset), and thus not detected. Subsequent NTM for this candidate single unit waveform detected all 3 spikes, and spike clustering determined that (ii) was part of the same single-unit cluster as spikes (i) and (iii). Spike rasters and peri-stimulus-time-histograms (PSTHs) for this same unit revealed that NTM detected many more whisker-evoked spikes than the standard method (Fig. 2b-c). In contrast, spontaneous spiking (before stimulus onset) was largely similar between NTM and the standard spike detection method (Fig. 2b,c).

Figure 2d–f summarize spike detection across 28 tetrode recordings in 7 mice. 72 single units were found using a standard fixed voltage threshold, and then NTM was used to re-detect spikes from these candidate units (mean firing rate across units: 2.96 ± 0.15 Hz and mean peak-to-peak amplitude: 79.68 ± 3.99 µV). Across these single-units, the number of spikes detected after NTM was greater than those detected with the standard voltage threshold. On average, 21.48% of spikes detected by NTM were missed by the voltage threshold method. This is approximately twice the number of spikes that were missed by NTM but detected with the voltage threshold (Fig. 2d). In addition, 21.85% of spikes were detected by both methods but classified into different clusters by the spike sorting step. This clustering error rate is within the expected range as assessed from simultaneous intracellular and extracellular recordings^29^ and is likely due to variability in automated clustering or manual curation procedures. Since this error is associated with clustering and not spike detection we discarded these spikes from subsequent analysis. NTM detected more spikes even after discarding misclassified spikes (Fig. 2e) (paired t-test p = 2.18e-6, n = 72 units; best-fit-line = 1.11x + 640.17).

Cluster quality after NTM was largely equivalent to the standard method, indicating that the additional spikes identified by NTM were similar in shape and amplitude to spikes identified by voltage threshold. To quantify this, we calculated the Mahalanobis distance of each spike to its cluster mean (spike waveform template), for spikes identified by standard voltage threshold and then subsequently by NTM. For each cluster, we compared the mean Mahalanobis distance across all spikes before and after NTM. These were not significantly different *(p* = 0.32*)* (Fig. 2f). Thus, NTM detects more spikes, with similar cluster quality, compared to the standard method.

### Mean spike waveform shape is conserved after NTM

**Figure 3:**
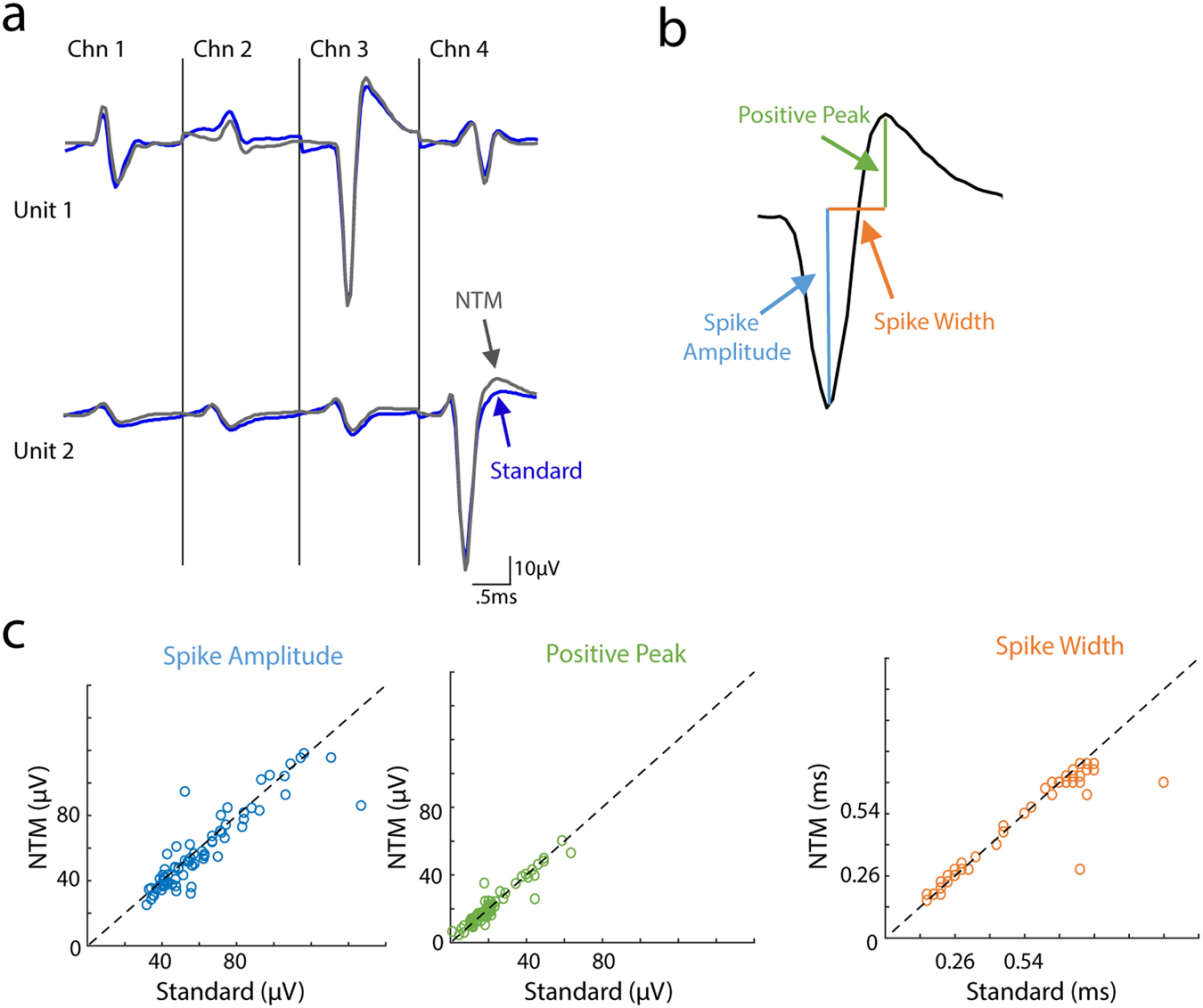
Mean spike waveform shape is conserved after NTM. **(a)** Mean spike waveforms of two example single-units with NTM and standard spike detection. **(b)** Schematic of the calculation of spike amplitude (current sink), positive peak (current source) and spike width (time difference between current sink and source). **(c)** Comparison across units for three spike waveform features.

The greater number of spikes assigned to each cluster after NTM suggests that NTM can detect spikes that were missed by the voltage threshold. If these ‘new’ spikes are not due to clustering errors then the mean spike waveform of each single-unit should be relatively unchanged after NTM. Figure 3a shows the mean waveform of two example single units before and after NTM, which have very similar overall waveforms. We quantified spike similarity by examining the spike amplitude at maximum negativity (reflecting the local current sink), positive peak (reflecting local repolarization) and spike width of the mean cluster waveform (Fig. 3b). We compared these three features for single-unit clusters before and after NTM. Across the population of 72 single units, no significant differences were found between NTM and standard method in negative spike amplitude, positive peak or spike width (paired t-test *p* = 0.38, 0.63 and 0.56 respectively), and the Pearson correlation was close to unity, with *r* = 0.93, 0.96 and 0.98 respectively (Fig. 3c). Thus, mean spike shape is not altered by NTM.

### Measuring sensory responsiveness and sensory tuning after NTM

Dense local population activity can cause failure to detect legitimate spikes (false negatives or type II errors) because of shadow periods using the standard voltage threshold method. This raises a critical concern that spiking evoked by the strongest stimuli will be systematically under-estimated, thus reducing measures of sensory responsiveness and artifactually flattening sensory tuning curves. In S1, most neurons within a single cortical column spike most strongly to one facial whisker that is anatomically matched to that column (termed the columnar whisker, CW)^30,31^. This topographical organization means that CW deflections will elicit more spikes among local neurons than deflections of surrounding whiskers (SWs), raising the possibility that type II errors are more common during CW deflections. This problem may be more acute in layer (L) 4, which has higher neuronal density and stronger multi-unit population responses than in L2/3. We tested whether improvements in spike detection from NTM were systematically different across whisker stimuli and across layers.

**Figure 4:**
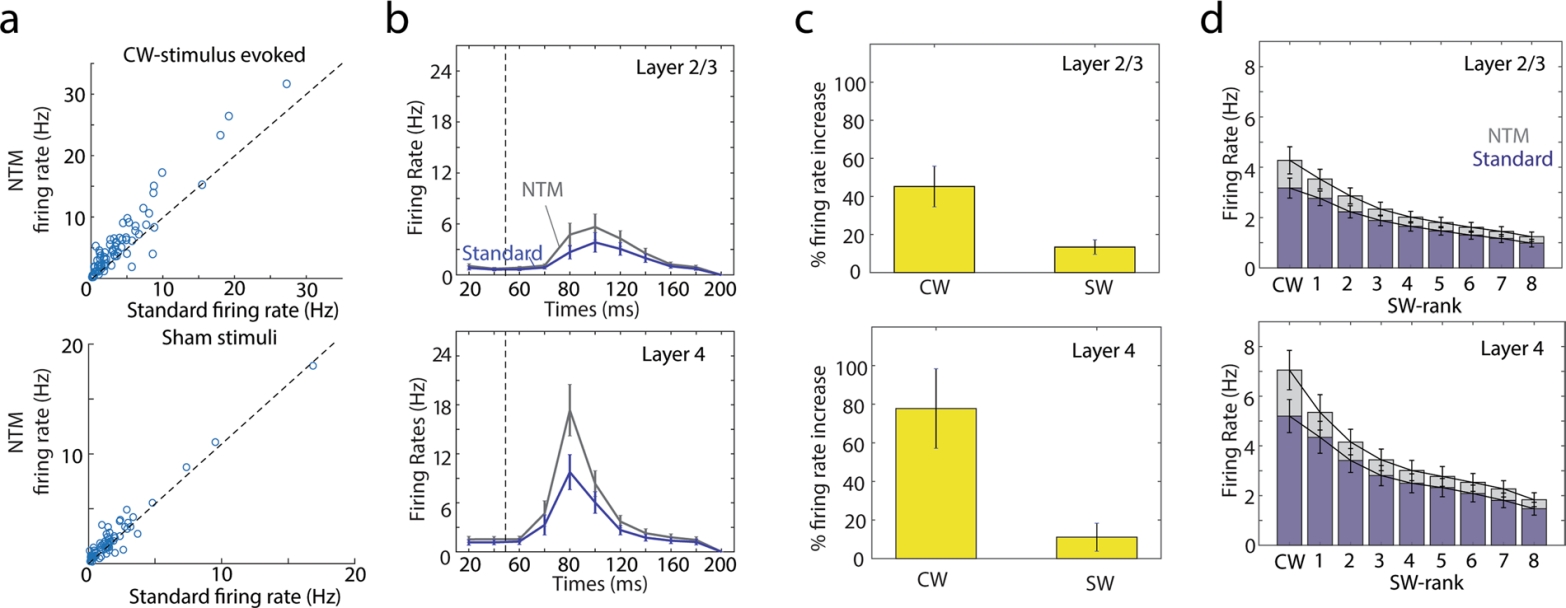
NTM improvements in spike detection are stimulus specific. **(a)** Top, comparison of measured columnar-whisker (CW)-stimulus evoked firing rates with NTM vs. the fixed voltage threshold method across all single-units. Bottom, same but for sham stimulation (i.e. spontaneous firing rates). **(b)** Average CW-stimulus peri-stimulus-time-histograms (PSTHs) across layer 2/3 and layer 4 single-units with NTM and the voltage threshold trigger. **(c)** Mean percent gain in measured CW- and surround-whisker (SW)-stimulus evoked firing rates with NTM relative to the standard voltage trigger. **(d)** CW and SW responses ranked by response strength for both NTM and the standard voltage threshold trigger. (**b**–**d**) All error bars are standard errors of the mean.

For each single unit cluster (n = 72), we compared the number of CW-evoked spikes (counted in a window 0–125 ms after stimulus onset) detected with standard and NTM methods. NTM detected significantly more CW-evoked spikes (paired t-test *p* = 0.0495) (Fig. 4a). In the same experiments, spontaneous firing rate was measured following sham whisker deflections. Spontaneous firing rate after sham stimuli was not significantly different between NTM and standard methods (*p* = 0.39) (Fig. 4a, bottom). Thus, sensory responsiveness (signal-to-noise ratio) was underestimated with the standard fixed threshold method. On average, CW-evoked firing rates were significantly greater with NTM for both layer 2/3 (2-way ANOVA, *p* = 1.9e-9) and layer 4 (*p* = 5.2e-7) populations (Fig. 4b). However, the greatest gain in detected spikes occurred for CW- over SW-evoked spikes, and for L4 over L2/3 (Fig. 4c). With NTM, L2/3 and L4 units had a 45.2% and 77.8% average gain in detected CW-evoked spikes, respectively, but only a modest 13.4% and 11.1% average gain for SW-evoked spikes (computed on average across 8 SWs). This suggests that standard spike detection will systematically underestimate the peak of whisker receptive fields. To test this, we calculated the whisker receptive field for each single unit after ranking SWs from strongest to weakest within each unit. The average whisker receptive field was sharper after NTM, because NTM improved spike detection most for the strongest stimuli (Fig. 4d). Thus, NTM appears to enhance detection of whisker-evoked spiking responses, especially for stimuli evoking strong spiking responses among nearby neurons.

### Source of newly detected NTM spikes

Spike events can be missed by the standard voltage threshold method for two reasons. First, the voltage amplitude of some spike events can be less than the user-defined detection threshold. Second, spikes can fall within the shadow period of a previous threshold crossing event. We tested which of these issues was primarily responsible for increased spike detection with NTM, under our experimental conditions.

For each NTM-detected spike that was missed by the standard threshold method, we determined whether the miss was due to a shadow period, or to spike amplitude that did not exceed detection threshold. For both L2/3 and L4 populations, most missed spikes were due to censoring by a shadow period. Shadowed spikes represented 60% of missed spikes in L2/3 and 70% of missed spikes in L4. Thus, most of the spikes missed by the standard voltage threshold method are due to local, temporally dense spiking near detection threshold.

### Benchmarking spike detection performance *in silico*

**Figure 5:**
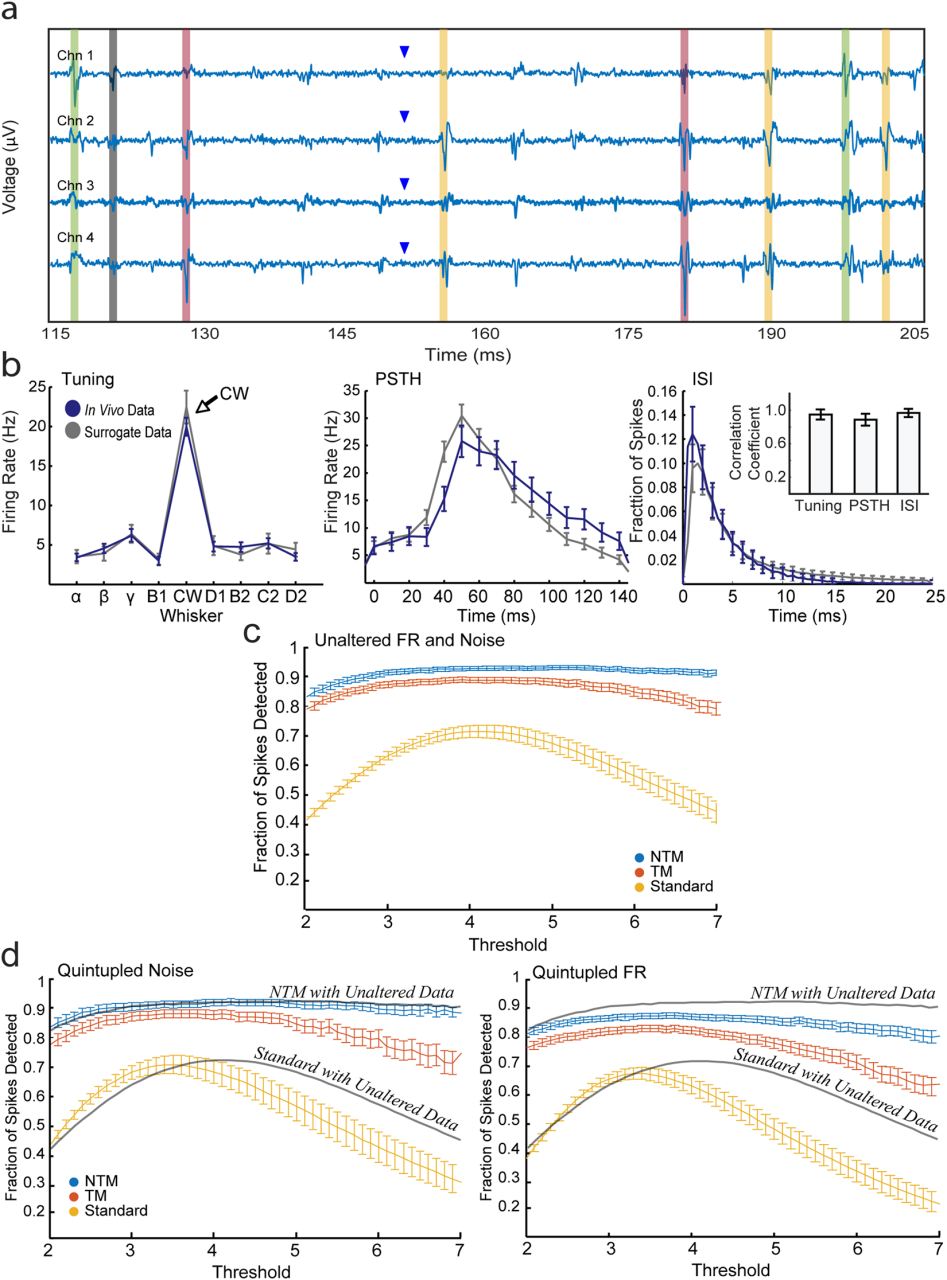
Performance of NTM on simulated voltage trace data. **(a)** An example segment of the surrogate voltage trace data. Regions in same color denote spikes from the same model neuron. Triangles represent whisker deflection onset time. **(b)** Tuning curve, PSTH, and ISI distribution computed across all spikes (including both multi- and single-unit spikes) in measured and simulated voltage data. Inset: Mean and standard deviation of tuning, PSTH, and ISI distribution correlation coefficients between model and *in* *vivo* single-units (n = 60). **(c)** A plot showing the effects of different voltage amplitude thresholds (expressed as number of standard deviations) on NTM, TM, and Standard spike detection methods. **(d)** Same plots as (**c**) but NTM, TM, and Standard methods were performed on surrogate data with quintupled noise and quintupled FR. Gray line represents plots from (**c**) for comparison.

To evaluate NTM performance with data for which ground-truth spike information is known, we applied it to computationally generated extracellular voltage traces built to resemble S1 activity during whisker stimulation. This surrogate data modeled background noise and the statistics of spiking activity from one representative tetrode recording, but with precise knowledge of the timing and cluster identity of each spike. For each simulated tetrode channel, we generated an independent Gaussian white noise signal with standard deviation equal to that recorded *in vivo*. We then probabilistically added spike waveforms from *n* actual recorded single unit clusters and 1 multi-unit cluster (consisting of voltage epochs that exceeded the fixed-voltage threshold but were not clustered into single-unit clusters) at specific times (Fig. 5a).

Spike times were chosen as follows. We modeled the spiking activity of each single-unit *i* as a Bernoulli random variable whose ‘success’ probability was time- and stimulus-dependent (i.e. a binary point process):

$${B}_{i,T}=Bernoulli({p}_{i}(t=T,W))$$

where *B*_*i*,*T*_ equaled 1 if the modeled neuron spiked at time *t* = *T* in a trial where stimulus *W* was delivered and equaled 0 otherwise. The function p_i_(t, W) describes the probability of model neuron *i* spiking as a function of time and stimulus identity. We computed p_i_(t, W) by convolving a 5 ms box filter with individual spike trains from single-unit *i* and then averaging this across trials where stimulus *W* was delivered. This generates a smooth PSTH that describes instantaneous spiking probability rather than firing rate. The function p_i_(t = T, W) was set to zero if model neuron *i* spiked 2 ms before time *T*, thus enforcing an absolute refractory period for single unit spiking. Multi-unit clusters are voltage spikes in real recordings that surpassed the fixed detection threshold, but which after spike clustering did not meet single-unit quality criteria. We modeled multi-unit activity as an inhomogeneous Poisson process:

$${P}_{T}=Poisson(\lambda (t=T,W))$$

where *P*_T_ was larger than 0 when the modeled multi-unit spiked at time *t* = *T* and was 0 otherwise. The function λ(t = T, W) is the mean of *P*_*T*_ and was analogous to p_i_(t, W) but computed from multi-unit spike trains. We chose a Poisson process to model multi-unit activity because 1) we sampled *B*_i,T_ and *P*_T_ at discrete 1-ms time-steps and there can be > 1 multi-unit spike within this time range (i.e. multi-unit activity lacks an absolute refractory period) and 2) the Poisson distribution is a good approximation to a sum of *n* independent Bernoulli random variables (single units) with different but small ‘success’ probabilities^32^ (spike probabilities).

At the time of each simulated spike (i.e., when *B*_*i*,*T*_ = 1 or *P*_T_ > 0), we selected one recorded tetrode spike waveform from the single- or multi-unit cluster, and linearly added it to the white noise signal. We added sub-millisecond jitter to the spike times via a uniformly distributed pseudorandom number ranging from 0 to 1 ms, to avoid sampling spike times only at integer 1 ms timesteps. This process was repeated for all simulated spikes across all modeled single-unit and multi-unit clusters, yielding surrogate tetrode voltage data with known spike times and cluster identities, and realistic variability in shape of individual spikes within each cluster.

We computationally generated 1000 trials of tetrode voltage data: 100 trials each that simulate deflection of 9 individual whiskers, and 100 trials that model sham stimulation (i.e., spontaneous activity). Mean firing rate across units was 2.86 ± 0.51 Hz. One example trial is shown in Fig. 5a. As expected, the computationally generated data replicated many aspects of stimulus-evoked neural activity in S1. Figure 5b shows the tuning curve (receptive field), PSTH, and ISI distribution computed across all spikes from the surrogate data, which closely match the corresponding *in vivo* data. The average Pearson correlation coefficient between modeled and measured single-unit tuning curves, PSTHs, and ISI distributions were close to unity, with r = 0.93, 0.87, and 0.95, respectively (Fig. 5b, inset).

### Template-based methods outperform the standard fixed-voltage threshold method *in silico*

We used the surrogate data set to compare performance of NTM, template matching without scaling (TM, which corresponds to a sliding dot product or cross-correlation) and standard fixed voltage threshold methods. Because we know the spike times of all modeled units, we can calculate the fraction of detected spikes for each model neuron relative to ground-truth data. No spike waveform clustering is needed to evaluate performance because the cluster identity of each spike is known. We discarded near-simultaneous spikes from analysis (defined as 2 separate spike events between single-units within 0.5 ms of each other; only 1.45 ± 0.11% of total spikes were classified as near-simultaneous) to avoid ambiguity in assigning a cluster identity to each detected spike.

We compared detection performance as a function of the fixed voltage threshold used for the first step of detection. The standard fixed threshold method performed best with a detection threshold of ~ 4 SD, where ~70% of spikes were detected. Performance deteriorated substantially with larger or smaller detection thresholds, highlighting the strong susceptibility to the experimenter’s choice of threshold. NTM and TM outperformed the fixed threshold method across all detection thresholds, achieving ~90% (NTM) and ~85% (TM) peak spike detection performance (Fig. 5c). Importantly, NTM and TM were much less dependent on choice of detection threshold. NTM performed well at almost all detection thresholds, while TM performance declined somewhat at >5 SD. The superior performance of NTM reflects the normalization process within NTM that enables detection of individual spikes smaller than the user-determined threshold, but with identifiable shape within and across tetrode channels. Thus, our data-driven approach to detect spikes (Fig. 1c) outperforms the standard fixed threshold method and largely eliminates the effect of the choice of a specific threshold for the initial round of spike detection.

We also tested the resilience of NTM and TM methods to higher recording noise and higher average firing rates. To do this, we generated two additional surrogate data sets, one with quintupled firing rates (mean 14.30 ± 2.53 Hz) and the other with quintupled noise. Both NTM and TM methods outperformed the standard method in both conditions, with NTM providing superior performance than TM (Fig. 5d). Thus template-based methods provide substantially more robust spike detection across a range of recording conditions.

## Discussion

We developed a spike detection algorithm based on template matching and tested its performance on extracellular recordings from multi-site silicon probe electrodes in S1 *in vivo*. Whisker receptive fields of S1 neurons are well characterized^30,31^. S1 is topographically organized so that the majority of neurons have their strongest response to the anatomically matched columnar whisker (CW). Thus, CW deflections evoke spatiotemporally dense spiking, a situation where spike detection is particularly challenging. We found that NTM substantially increased the number of stimulus-evoked spikes detected after CW deflection, and showed a weaker improvement for SW-evoked spikes, which are fewer and spatiotemporally less dense in S1. The greatest gain in detected spikes was observed in L4, where spiking probability and spike synchrony are greatest^19,33^. Thus, NTM provides a more accurate estimate of sensory tuning and sensory-evoked firing rate than standard fixed-threshold spike detection in S1. This same improvement is also expected in other topographically organized cortical maps, due to improved spike detection by NTM during spatiotemporally dense neural activity.

Most spikes missed by the standard fixed voltage threshold method were due to the shadow period enforced after preceding voltage threshold crossing events. Thus, detection by a fixed voltage threshold can yield many type II errors in spike detection (missed spikes), which compromise signal-to-noise and sensory tuning measurements. Because these mostly reflect shadow periods from prior low-amplitude spikes or near-threshold recording noise, they could in principle be avoided by using electrodes that better isolate a smaller number of nearby units (i.e., have a smaller effective recording radius). However, many modern recording devices, like silicon probes, are designed to maximize the number of recorded units, and thus include substantial non-sortable, low-amplitude spikes and recording ‘hash’ when local neural populations are strongly activated. NTM provides a method for improving spike detection with these devices, under conditions when strong and temporally dense network activity exists. We show that this enables more accurate measurement of stimulus-evoked spikes and sensory tuning. In addition, NTM performance is much less dependent on the experimenter’s choice of spike detection threshold, or on recording noise levels, and thus is likely to be more robust across experimental conditions.

NTM and its data-driven approach for choosing detection thresholds from template-filtered voltage traces is similar to some prior spike detection methods^9,10^. One difference over these prior methods is that NTM does not require computing the noise covariance matrix and its inverse, which are computationally intensive steps. NTM computes a sliding cosine similarity rather than the cross-correlation between the whitened voltage signal and template, making NTM computationally and mathematically simpler. See ref.^12^ for a similar approach. This also allows NTM to be easily implemented in applications that perform template matching in real-time (e.g. CED Spike2 and ref.^11^).

We did not directly compare NTM performance with other available model- or filtering-based spike detection methods^9–11,13–17^. This is because our goal was not to establish any one absolute superior spike detection method, but instead to identify errors common to the classic fixed threshold method and show that even the simplest implementation of template matching provides substantially improved spike detection. The relative performance of different algorithms will depend strongly on the specific spatiotemporal characteristics of neural activity and structure of background noise, which vary across brain areas and recording systems. Overall, our results indicate that template-based spike detection methods such as NTM can greatly improve quantification of single-unit spiking activity, especially in topographically organized areas like primary sensory cortex.

## Methods

The *in vivo* physiology data that are analyzed in this paper were collected as part of a larger study (ref.^34^) These data are from *Experiment 2* of that study.

### Surgical preparation and recording electrode placement *in vivo*

All animal procedures were approved by the UC Berkeley Animal Care and Use Committee and meet NIH guidelines. Male C57BL/6 mice (age: P28-45) were used. Mice were anaesthetized with urethane and chlorproxithene (1.3 g/kg and 0.02 mg in 10 mL saline). Body temperature was maintained at 36.5 °C using a feedback-controlled heating pad (FHC, 40-90-8D). Anesthetic depth was assessed via toe pinch and supplemental urethane (10% of initial dose) was provided as needed. The skull was exposed, cleaned and a stainless steel head-post was implanted. A 2 mm craniotomy was made over S1 (coordinates: 1.5 mm rostral, 3.3 mm lateral of bregma). The target column (the C1 or D1 whisker column) was localized using receptive field mapping of multi-unit activity in L4, recorded with a tungsten microelectrode.

A silicon laminar probe (NeuroNexus, 32 channel, 1 shank, with Poly2 or Poly3 channel geometries (A1x32-Poly2-5mm-50s-177 and A1x32-Poly3-6mm-50-177-A32) was then inserted radially into the target column via a small durotomy. The probe was slowly advanced until the deepest recording pad was in L4. Simultaneous L2/3 and L4 recordings were made at this depth. L2/3 and L4 were defined by microdrive depths as 100–417 and 418–587 µm below the pia^35^.

### Whisker stimulation

Calibrated deflections were applied independently to a 3 × 3 grid of whiskers, centered on the columnar whisker for the recorded column. Stimuli were controlled using custom software in Igor Pro (Wavemetrics). Each whisker was trimmed and inserted into a glass tube carried on a piezoelectric bender actuator. The piezo was positioned to deflect the whisker at 5 mm distance from the face. Each whisker was deflected rostrocaudally with triphasic waveform that was shown previously to optimally drive S1 neurons and captured most of the evoked response variance in S1 (first common filter)^36^. The whisker deflection waveform was 40 ms duration, 300 μm peak amplitude, and had a mean frequency content of 53 Hz.

The stimulus set included 18 different single-whisker deflections (9 whiskers with peak deflection amplitude in either the rostral or caudal direction). Additional 2-whisker combination stimuli were also applied but were not analyzed here. All stimuli were randomly interleaved at 0.6 s inter-stimulus interval, yielding an overall average deflection rate for any whisker of 3 Hz. Each recording was 3.5–6 hours. Sham stimuli (blank trials in which no whisker was deflected) were randomly interleaved with other whisker stimuli to quantify spontaneous spiking.

### Data acquisition and preprocessing

Recordings were amplified and bandpass filtered (Plexon Instruments PBX2/16sp-G50, × 1,000 amplification, 0.3–8 kHz bandpass) and digitized at 31.25 kHz. Noise was reduced by common average referencing^25^; the average voltage signal across all electrodes in the brain was calculated and subtracted from the signal in each electrode. Poly2 electrodes have two vertical rows of recording pads with 50μm inter-pad spacing. These pads were divided into groups of 4 spatially adjacent pads, which were treated as the 4 channels of one tetrode. Adjacent tetrodes used neighboring, non-overlapping sets of pads, and were 50 μm apart. Poly3 electrodes were also divided into 4-channel tetrode groups. Because Poly3 electrodes have three vertical rows of recording pads, they contain 6 pads within each 50μm depth range, and thus there are 15 possible tetrode configurations for each 50μm depth range (i.e., 6 choose 4 configurations). We selected just one tetrode for each 50μm depth range by choosing the 4 pads in each 50μm depth range that exhibited the best signal-to-noise ratio. Signal-to-noise ratio was calculated independently for each electrode pad as the kurtosis (i.e. “peakedness”) of the voltage signal across time: ${{\rm{k}}}_{{\rm{c}}}={\rm{E}}[{({\rm{v}}{({\rm{t}})}_{{\rm{c}}}-{\rm{E}}[{\rm{v}}{({\rm{t}})}_{{\rm{c}}}])}^{4}]$. Adjacent tetrodes on Poly3 electrodes used non-overlapping sets of pads. Spike detection and sorting were performed on each tetrode separately.

### Spike sorting

In the standard fixed-threshold method, negative-going spikes were detected using an amplitude threshold (2.8–3.2 s.d. of noise floor), with a shadow period of 0.66 ms after each threshold-crossing. We chose a 0.66 ms shadow period because this was the smallest value that prevented single spike events as being detected more than once. Detected spikes were then clipped (1.5-ms waveforms) for spike sorting. Isolatable single-units were labeled through manual inspection and had to satisfy the following criteria: <0.5% refractory period violations (defined as inter-spike interval <1.5 ms) and <30% estimated missed spikes (based on Gaussian fit of detected spike amplitudes relative to the voltage amplitude detection threshold). The mean spike waveform of each single-unit was then used as a template for NTM spike detection. As in the standard method, a shadow period was enforced after each voltage segment detected as a spike (0.66 ms). Clustering was then redone as in the standard method. Details of the NTM method are given in the Results section.

All of the spike sorting used the open-access software UltraMegaSort2000^27^ (ums2k), implemented in Matlab. In the standard method spike detection, alignment, clustering and manual curation were all done with the functions provided in the ums2k package. For NTM, spike detection was done with custom software in Matlab (the NTM algorithm is described in detail in the results section) and spike alignment, clustering and manual curation were all done with the ums2k software package. Note that NTM can be implemented with any clustering algorithm.

## Data Availability

Code to perform the NTM method will be made available upon request via email to the corresponding author.
