# Author Correction: A normalized template matching method for improving spike detection in extracellular voltage recordings

**DOI:** 10.1038/s41598-019-53689-y

**Published:** 2019-11-19

**Authors:** Keven J. Laboy-Juárez, Seoiyoung Ahn, Daniel E. Feldman

**Affiliations:** 1 Department of Molecular and Cell Biology and Helen Wills Neuroscience Institute, University of California Berkeley, Berkeley, CA 94720 USA; 2 Present Address: Department of Organismic and Evolutionary Biology and Center for Brain Science, Harvard University, Cambridge, Massachusetts, USA

Correction to: *Scientific Reports* 10.1038/s41598-019-48456-y, published online 19 August 2019

The original version of this Article contained an error in Affiliation 1, which was incorrectly given as:

‘Depatment of Molecular and Cellular Biology and Helen Wills Neuroscience Institute, University of California Berkeley, Berkeley, CA, 94720, USA’

The correct affiliation is listed below:

‘Department of Molecular and Cell Biology and Helen Wills Neuroscience Institute, University of California Berkeley, Berkeley, CA, 94720, USA’

This error has now been corrected in the HTML and PDF versions of the Article.

